# Asthma in changing environments - chances and challenges of international research collaborations between South America and Europe - study protocol and description of the data acquisition of a case-control-study

**DOI:** 10.1186/1471-2466-10-43

**Published:** 2010-08-18

**Authors:** Anja Boneberger, Katja Radon, Jennifer Baer, Leonie Kausel, Michael Kabesch, Daniel Haider, Rudolf Schierl, Rüdiger von Kries, Mario Calvo

**Affiliations:** 1 Institute and Outpatient Clinic for Occupational, Social and Environmental Medicine, Hospital of the Ludwig-Maximilians-University Munich, Ziemssenstrasse 1, 80336 Munich, Germany; 2 Facultad de Bioquímica, Universidad Austral de Chile, Valdivia, Chile; 3 Center for Paediatrics, Clinic for Paediatric Pneumology, Allergology and Neonatology, Hannover Medical School, Carl-Neuberg-Str. 1, 30625 Hannover, Germany; 4 Institute for Social Pediatrics and Adolescent Medicine, Department of Epidemiology, Ludwig-Maximilians-University Munich, Heiglhofstrasse 63, 81377 Munich, Germany; 5 Facultad de Medicina, Universidad Austral de Chile, Valdivia, Chile

## Abstract

**Background:**

Asthma in children is an emerging public health problem in South America. So far, research in this part of the world is limited. This paper presents the methodology and description of the data acquisition of an asthma case-control study conducted in the Central South of Chile.

**Methods/Design:**

A hospital-based case-control study about asthma (188 cases, 294 controls) in children (6-15 years) was carried out in Valdivia, Chile between November 2008 and December 2009. Data on asthma risk factors were collected by computer-assisted personal interview using validated questions from e.g. ISAAC phase II. Data on household dust exposure (endotoxin, allergen analyses), skin prick tests to most common allergens, stool examinations for parasitic infection, and blood samples (total IgE, genetics) were collected. Additionally, 492 randomly chosen blood donors were recruited in order to assess allele frequencies in the population of Valdivia.

**Discussion:**

Overall 1,173 participants were contacted. Response was 82% among cases and 65% among controls. Atopic sensitization was high (78% among cases, 47% among controls). Cases had a statistically significantly (p < .0001) increased self-reported 12-month prevalence of symptoms of rhinitis (82% vs. 51%) and wheeze (68% vs. 16%). The study is well placed to address current hypotheses about asthma and its correlates in the South American context. Results of this study might help develop novel, innovative and individualized prevention strategies in countries in transition with respect to the South American context.

## Background

Asthma in children has been described as a new public health challenge for South American countries [1-3]. According to the ISAAC (International Study of Asthma and Allergies in Childhood) study, the prevalence of asthma in some Latin American countries (Brazil, Peru, Costa Rica, Paraguay, and Uruguay) has increased to that of industrialized and known high prevalence countries [2].

Most South American countries have undergone profound political, economic, demographic, and epidemiologic changes which have lead to the adoption of a "western" lifestyle, including urbanization, modernization, and change of living conditions and dietary habits [4,5].

The increase of asthma has been explained mainly in European countries by the hygiene hypothesis [6]. This hypothesis states that unhygienic contact in early life, e.g. family size and day care attendance, and infections in early life might be protective against allergic diseases. The applicability of the hygiene hypothesis for Non-European countries has been challenged as asthma in South American inner cities increased which are characterized by poor housing, overcrowding and dirty environment [3,7-9].

The study presented here, VERMEE (**V**aldivia **E**ncont**R**ando **M**únich - **E**studio **E**pidemiológico), is a research collaboration between Chile and Germany. The study aims to assess the impact of genetic and environmental factors in a South American country of epidemiological transition by

- measuring the role of specific microbial exposure (i.e. endotoxins) and nutritional factors

- elucidating the molecular underpinnings of asthma. Most of molecular based studies have mainly been confined to the genetics of European and North American populations excluding populations from developing countries.

This paper presents the methodological aspects and description of the data acquisition of this case-control study conducted in a predominantly rural region in the Central South of Chile.

## Methods/Design

### Chile - a country in epidemiological transition

Chile (16 million inhabitants) is among the most developed countries in South America: Poverty rate has recently declined from 38.6% in 1990 to 13.7% in 2006 [10].

Since 2010, it is the first South American country to be member of the Organisation for Economic Co-Operation and Development (OECD) [10]. In 2009, Chile had the highest (worldwide: 44^th ^place) Human Development Index (HDI), a composite index to measure human development proposed by the United Nations Development Programme (UNDP), among South American countries. Infant mortality rate has constantly decreased to 7.7 per 1,000 live births, comparable to the one in the United States and only slightly higher than in Europe [11,12]; adult literacy rate has reached 96.5% in 2007 (1990: 78.3%) [13]. Furthermore, housing conditions have improved: potable water coverage nears 100% in urban areas and exceeds 90% in more densely populated rural areas; 96.1% of households have electricity coverage [14].

Chile is currently in a state of epidemiological transition with an incidence of communicable diseases at the lowest level ever and non-communicable diseases (diabetes, asthma etc.) on the rise [5,14,15].

### Study site

The study was based in Valdivia (153.000 inhabitants), the capital of the Region de los Ríos, in the Central South of Chile. The main economic sectors are forestry and agriculture and one third of the population lives in rural areas [16].

### Study population and study design

A case-control study including 188 cases and 294 controls was conducted between November 1, 2008 and December 15, 2009 among children and adolescents aged 6-15 years. The sample size results in a power of 84% to detect an Odds Ratio (OR) of 0.5 if exposure prevalence was 30% among controls. However, prevalence of exposure had to be estimated as no previous data existed.

Two controls were frequency-matched on age and sex per case. Frequency-matching aims to have equal proportions of potential confounding factors among cases and controls and is considered to be more efficient than individual matching [17].

Cases were recruited from two sources, the public Hospital Base Valdivia [18] and the Consultorio Externo. The Hospital Base Valdivia is the only public hospital in Valdivia. The Consultorio Externo (Avenida Francia, Valdivia) is one of three outpatient clinics for primary health care in Valdivia covering about 38,000 people from neighbouring parts ('barrios') of Valdivia [19].

A "consultorio" is an institution of primary care treating publicly insured patients with general medicine and paediatrics (no emergencies). Both institutions are state-run.

In the Hospital Base Valdivia, controls were recruited from general medicine, internal medicine, paediatric surgery, dentistry, neurology, orthopedics/traumatology, and gynecology. In the Consultorio Externo controls were recruited from the general consultation, including e.g. general medicine, internal medicine, and paediatrics.

### Inclusion criteria, main outcomes and case definition

All cases with a doctor's diagnosis of asthma in the patient registry were eligible. In Chile, asthma is mainly clinically diagnosed and treated. From 2006 onwards, Chile has adopted World Health Organization's GINA (Global Initiative for Asthma) guideline [20,21].

Cases and controls were included if they

- lived in the region of Valdivia

- had lived throughout their life in an either urban or a rural setting

- were accompanied by a parent/legal guardian

- were born in Chile

- were not physically/mentally disabled

Controls with self-reported physician's diagnosis of asthma were excluded.

### Non-eligible patients and non-responders

Basic sociodemographic characteristics of (i.e. date of birth, sex, case or control status, and reason for non-participation) patients not meeting the inclusion criteria (non-eligible) or refusing to participate (no interest, no time, general doubts about the study, other) were recorded.

### Population-based blood donors

Additionally to the case-control-study, blood samples were taken from 492 randomly chosen blood donors from the blood bank in Valdivia (Banco de Sangre, Hospital Base Valdivia) and their DNA was extracted and analysed in order to assess allele frequencies in the general population of Valdivia without the risk of population stratification by case or control status. This had to be done as no previous study on the allele frequencies was conducted in the population of Valdivia.

Inclusion criteria for the blood donors were age ≥ 18 years and written informed consent to participation in the study. Blood donors were asked for 5 ml of blood. Basic sociodemographic information (i.e. sex, age, and reason for donation) was recorded.

### Data collection and study instruments

**Figure 1:**
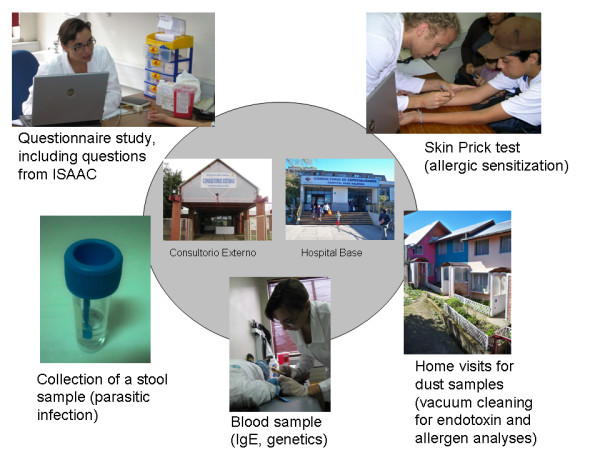
**Description of the study parts done in Valdivia, Chile**.

Participants were free to choose on which study parts they wanted to participate. The fieldwork and study parts of VERMEE are presented in Figure 1.

#### 1. Questionnaire

The computer-assisted personal interview (CAPI) questionnaire used in VERMEE was based mainly on the ISAAC phase II questionnaire [22], the ALEX [23], and the CAT [24] studies including questions about the following: Asthma symptoms and symptoms of atopic diseases, presence of asthma; infections and vaccinations, family size, family history of asthma, living conditions (urban, rural), contact with animals, and indoor environment: environmental tobacco smoke exposure, pets, dampness and mould, cooking and heating fuels. Additional questions about nutrition status from German questionnaires were translated into Spanish with back-translation according to a standardized protocol and were pilot tested.

#### 2. Anthropometry

Children were weighed in light clothing and without shoes. Height was assessed using a stadiometer. Weight was assessed using a standard scale.

#### 3. Skin Prick Test (SPT)

Skin prick testing to the following allergens was done according to the ISAAC protocol [25]: cat, dog, cockroach, house dust mites (*Dermatophagoides pternonyssinus, Acarus sirus*), mould (*Alternaria tenius*), trees, local grass mix, and weed mix (Laboratorio Leti, España). Mean wheal diameter was measured using both the longest and orthogonal diameters, recorded to the nearest 0.5 mm. A test was considered positive if the wheal size was at least 3 mm greater than the saline control 15 minutes after pricking the allergen into the forearm using a lancet for skin prick tests (Laboratorio Leti, España) [26].

#### 4. Stool sample

Stool samples were examined at the university laboratory for helminths/parasites (i.e. *Endolimax nana*, *Blastocystis hominis*, *Entamoeba coli*, *Giardia lamblia*) using microscopy.

#### 5. Blood samples and DNA extraction

Blood samples were collected by venipuncture and were analysed for total IgE at the Hospital Base Valdivia. 5 ml of whole blood was also collected into EDTA sample tubes for DNA extraction. Extraction of human DNA was done through a salting out technique from whole blood [27]. The whole blood samples were stored at 4°C and processed within one to five days at the laboratory of the Facultad de Bioquímica, Valdivia. In brief, whole blood was treated with lysis buffer, centrifuged, the cell sediment was washed twice in buffer to obtain nucleated cells. The pellet was resuspended in a buffer containing Sodium Dodecyl Sulfate (SDS) to open the cells and incubated with proteinase K to digest proteins. The genomic DNA was obtained by addition of high concentration of salt (salting out) and the addition of ethanol. The DNA precipitate was washed in 70% ethanol, air-dried, resuspended in TE buffer and stored at 4°C. For genotyping, DNA was shipped at room temperature to the Hannover Medical School, Hannover, Germany.

#### 6. Dust samples

Dust samples were collected using a standard vacuum cleaner (Thomas hoover (TH -1850) with ALK-device (ALK Allergologisk Laboratorium, Copenhagen, Denmark). The study participants' living rooms were vacuum-cleaned for four minutes on smooth surface and/or carpet according to a standardized protocol. Dust was sieved to separate fine particles from fibres and large particles and then stored at room temperature until shipping to the laboratory of the Institute and Outpatient Clinic for Occupational, Social-, and Environmental Medicine, Hospital of the Ludwig-Maximilians-University Munich, for analyses. Dust samples were analyzed for endotoxin using a chromogenic Limulus assay (kinetic-QCL, Lonza, Basel, Switzerland) and a validated laboratory protocol [28]. Aeroallergen content (house dust mites (Der p1), cockroach (Bla g2), cat (Fel d1), dog (Can f1), rat (Rat n1) and mouse (Mus m1) was determined with a MARIA kit (Indoor Biotechnologies, Charlottesville, USA) using a multiplex bead based assay.

### Overview of statistical plan of analysis

Statistical analyses will be conducted according to a conceptual framework. Cross-tabulation will be used to visualize bivariate distributions of categorical predictors and outcomes. Exposure data will be compared between cases and controls. In order to assess the association between exposure and outcome, logistic regression models will be calculated. All analyses will be adjusted for the frequency-matching variables age and sex. Non-responders and responders will be compared regarding age using Wilcoxon-Rank-Sum-Test and sex using Chi-Square-Test to assess whether non-responders differ significantly in age and sex from responders.

### Genetic

Genetic analyses will be performed using the SAS genetics package, assessing the effects of genetic variation in single gene analyses using Chi-square statistics as well as Cochran-Armitage and two-way analyses of covariance (ANCOVA) trend tests. Haplotype structures were analyzed in haploview. Heterogeneity of association between the two strata (Chile and Germany) will be assessed by a weighted linear combination test using the results of an additive-effects-only regression analysis within each stratum. Ex-linked markers are analysed by fitting an additive effects-only logit model which equates the risks of male hemizygotes with female homozygotes. Gene environment analyses will be performed using the Botto Khoury approach as previously described [29]. To adjust for multiple testing, the Benjamini-Liu method which control subjects the false discovery rate (FDR), within a pre-defined family of tests, will be used.

### Ethical considerations

Ethical approval for the VERMEE study has been obtained from the ethics committees of the Hospital of the Ludwig-Maximilians-University of Munich and the Medical Faculty of the Universidad Austral de Chile. Written informed consent to participate in the study was obtained from both parents and children/adolescents. The parent/legal guardian of each child was provided with a copy of all laboratory results and, if necessary, treatment recommendations were made by the study team which reviewed each case.

### Safety considerations and quality assessment

All participants' data were entered in pass-word protected (ACCESS-) databases which were reviewed on an ongoing basis. E-Mails and phone conferences were held regularly to facilitate the team work. Additionally, field visits were done by the project partners to survey the ongoing study. Copies of the databases were saved on external hard disks. All databases will be kept for ten years and archived on an external drive.

### Funding

The study has been funded by the German Research Collaboration with Developing Countries Programme (DFG/BMZ), the German Academic Exchange Service (DAAD), and the German Federal Ministry of Education and Research (BMBF).

### Description of the data acquisition

A total of 1,173 (424 cases, 749 controls) were contacted between November 1, 2008 and December 15, 2009. There were 382 participants (32% of all patients contacted) not fulfilling the study criteria. Response was 82% (238/291) among cases and 65% (327/500) among controls. For all subsequent analyses, 482 patients (188 cases, 294 controls) with complete information on the questionnaire and skin prick test will be included. Details of the patient recruitment and response to the study parts are given in Table 1.

**Table 1:** Recruitment of the study population

	Cases		Controls	
	N	%	N	%
Patients contacted	424	100	749	100
Not eligible	133	100	249	100
Moved from urban to rural area or vice versa	62	46.6	143	57.4
Without parent/legal guardian	19	14.3	33	13.3
Place of living outside Valdivia region^a^	16	12.0	40	16.1
Asthma diagnosis not confirmed by a physician	29	21.8	NA	NA
Not born in Chile	1	0.8	3	1.2
Mentally/physically disabled	4	3.0	2	0.8
Control patient reporting diagnosis of asthma	NA	NA	24	9.6
Other	2	1.5	4	1.6
Eligible study population	291	100	500	100
Non-responder	53	100	173	100
No interest	21	39.6	90	52.0
No time	22	41.5	58	33.5
General doubts	9	17.0	12	6.9
Other	1	1.9	13	7.5
- Study population with complete interview and SPT data	188	100	294	100
**Participation in additional study parts:**				
Stool samples	138	73.4	137	46.6
Blood samples	163	86.7	245	83.3
Dust samples^b^	112	59.6	188	63.9

NA indicates not applicable

^a ^Indicating that people lived more than 100 km away from Valdivia

^b ^Dust samples from home visits

In the non-responder analyses participants and non-responders did not differ statistically significantly in sex (p-value: 0.99) and age (p-value: 0.32) (data not shown).

Of the asthma cases, 42% (71/177) were diagnosed in the first year of life. Most of the asthma cases (74%) were diagnosed with bronchoconstriction (data not shown).

Details of diseases presented by controls are shown in Table 2. The most frequent reasons for the controls were common cold (20%), orthopaedic problems (13%) or controls underwent surgery (e.g. phimosis, undescended testicle) (13%).

**Table 2:** Diseases covered by controls (N = 294) of an asthma case-control study from Valdivia, Chile

	N	%
Common cold (partly including fever)	59	20.0
Orthopedics (including accidents)	38	12.9
Surgery	37	12.6
Atopic diseases (including dermatology)	33	11.2
Otolaryngology	24	8.2
Gastroenterology	24	8.2
Dental check	17	5.8
Neurology	15	5.1
Routine check	12	4.1
Infectious diseases	9	3.1
Psychological problems	7	2.4
Endocrinology	5	1.7
Gynaecology	5	1.7
Ophtalmology	3	1.0
Nephrology	3	1.0
Vaccination programme	2	0.7
Missing	1	0.3

Cases and controls were comparable with regard to the matching variables age and sex. Some 78% of the cases, and about half of the controls (47%) showed at least one atopic sensitization in the skin prick test (Table 3). 12-month prevalence of rhinitis (82% vs. 51%) and wheeze (68% vs. 16%) were statistically significantly (p-value: <.0001) higher among cases (Table 3).

**Table 3:** Sociodemographic characteristics of the study sample

% (N)	Cases (N = 188)	Controls (N = 294)
Mean age in years (SD)	10.6 (2.6)	10.7 (2.7)
Boys	99 (52.7)	157 (53.4)
Residence in urban area of Valdivia	150 (80.7)	252 (85.7)
Atopic sensitization^a^***	147 (78.2)	138 (46.9)
At least one parent with atopic disease***	65 (34.6)	56 (19.1)
At least one parent with university attendance	38 (20.3)	59 (20.1)
Total IgE > 100 IU/ml***	104 (64.2)^c^	108 (44.1)^d^
Parasitic infection^e^	26 (18.8)^f^	28 (20.4)^g^
**12 months prevalence of:**		
- wheeze***	126 (67.7)	48 (16.4)
- rhinitis***	154 (81.9)	150 (51.0)
- atopic dermatitis symptoms	45 (24.3)	56 (19.2)

^a ^Positive skin prick test at least one allergen

^b ^Atopic disease indicates self-reported asthma or rhinoconjunctivitis

^c ^N available: 162

^d ^N available: 245

^e ^Parasitic infection to *Endolimax nana*, *Blastocystis hominis*, *Entamoeba coli*, and *Giardia lamblia*

^f ^N available 138

^g ^N available 137

*** chi ^2 ^< 0.0001

SD standard deviation

Some 56% of the 492 blood donors were male. The age range was 18-63 years (median: 30 years). Main reason for blood donation was a regular blood donation (49%).

## Discussion

Asthma in children is a growing public health challenge in South American countries. The VERMEE (**V**aldivia **E**ncont**R**ando **M**únich - **E**studio **E**pidemiológico) study, a case-control study conducted in the Central South of Chile, is one of the first studies assessing factors associated with disease status in this country in epidemiological transition using objective measures. Information about environmental and genetic factors and their association with asthma using objective measures is to our knowledge so far limited for South American countries [30,31].

The main objective of this study is to assess the applicability of the hygiene hypothesis in the Chilean context. Firstly, because Chile is a country in epidemiological transition undergoing rapid change and adopting the Western lifestyle [32]. Secondly, the global application of the hygiene hypotheses with its "established" asthma risk factor on global level has been questioned for low- and middle-income countries [33], including Latin American countries [34,35].

### Strengths and Limitations of the study

Response was higher among cases (82%) than among controls (65%). Non-responders among controls may have had less interest in the time-consuming study parts, or children were afraid of the skin prick testing or the venepuncture for collection of the blood sample. However, non-responders and study participants in the study did not differ significantly with respect to age or sex.

The overall participation in the different study parts was high: More than 80% of the study participants' gave a blood sample. However, controls were less motivated to return stool samples (47%) than cases (74%).

The acceptance of home visits (60% among cases, 64% among controls) was good and in the range of other studies [23]. About 16% of the home visits could not be realized because study participants could not be contacted (participants were not at home during an appointed visit, or telephone number was wrong). In Chile, the majority of people are using pre-credited sim cards for their mobiles, but most of them do run out of credit by the end of the month and hence it was impossible to get hold of them by the study team.

Another 10% of the home visits were realized, but there was not enough dust collected for analyses. In contrast to central Europe, most of the floors in Chile are smooth and are cleaned on a daily basis making it difficult for the study team to collect enough dust.

The selection of an adequate control group is the biggest challenge in any case-control study [36]. In order to find a comparable control group, we restricted our inclusion criteria to children that had lived throughout their live in an either urban or rural setting. Both cases and controls were recruited from public, state-run institutions (Hospital Base Valdivia, Consultorio Externo) which cover about 70% of the publicly-insured Chilean population [37].

Almost half of the control group were sensitized to at least one allergen. This high number of atopic patients suggests that parents suspecting allergic symptoms in their child were more likely to participate in the study which could have biased our results. On the other hand, as shown from results from the ISAAC study, general asthma symptom prevalence is generally high in Chile [1].

The questionnaire instrument was used in face-to-face interview (CAPI) potentially leading to an interviewer bias ("social desirability") or interpreting the questions by the interviewer. Interviewers were, therefore, intensively trained in order to avoid interviewer bias. Reading the questions to the study participants proved to be more efficient. This method helped to minimise missing values and to facilitate comprehension of the study questions. Albeit in our study sample, the education level was comparatively high - one fifth of the parents reported college education - which is slightly higher than recently published [38], the general low level of education leaves many Chileans as functional illiterates. This means that people technically know how to read and write, but are unable to understand the most basic written text.

The field phase of the VERMEE study required extensive co-ordination of the large study team in the study centres in Chile (Valdivia) and Germany (Munich, Hannover) involving experience from different disciplines (epidemiology, pediatrics, allergology, genetics). E-Mail contacts on a daily basis and Skype conferences on a regular basis were held to keep study centres up to date and to facilitate study coordination. Additional field visits by the study coordinators supervised the ongoing study. Cultural differences and the difference in time due to the large distance were mastered successfully.

The biggest chance of this international study was the way of capacity-building (three medical doctor theses, one master thesis, one PhD thesis) and the building of increased research capacity. Administrative and language barriers were low, and the cultural diversity enriching.

This study aims to clarify the gene-environmental interactions that lead to the increase in paediatric asthma in a country in epidemiological transition - a phenomenon most of South American countries are currently facing. We expect that the results of this study might help to identify public health interventions and establishing targeted interventions for the South American countries. The cultural and scientific exchange resulting from this study is stimulating and enriching - the exchange of knowledge in different cultural environments is promising for future studies.

## Competing interests

The authors declare that they have no competing interests.

## Authors' contributions

KR and MC conceived and designed the study. KR helped to draft the manuscript, RS participated in the design of the study, developed the sampling strategy for dust samples, and is responsible for the endotoxin and allergen analyses of the dust samples, RvK participated in the design of the study, and provided epidemiologic advice, MK carried out the genetic analyses and is responsible for the genetic study part. LK carried out the DNA extraction, JB and DH participated in the data acquisition. KR, MC, and AB coordinated the study. AB drafted the manuscript, data management, and statistical analyses. All authors have read and approved the final manuscript.

## Pre-publication history

The pre-publication history for this paper can be accessed here:

http://www.biomedcentral.com/1471-2466/10/43/prepub
